# Supplementary material for: Preexisting memory CD4 T cells in naïve individuals confer robust immunity upon hepatitis B vaccination
Source: eLife. 2022 Jan 25;11:e68388. doi: 10.7554/eLife.68388 (PMC8824481; doi:10.7554/eLife.68388)
Supplement: Transparent reporting form [file elife-68388-transrepform1.docx]

***eLife’s* transparent reporting form**

We encourage authors to provide detailed information *within their submission* to facilitate the interpretation and replication of experiments. Authors can upload supporting documentation to indicate the use of appropriate reporting guidelines for health-related research (see [EQUATOR Network](http://www.equator-network.org/%20)), life science research (see the [BioSharing Information Resource](https://biosharing.org/)), or the [ARRIVE guidelines](http://www.plosbiology.org/article/info:doi/10.1371/journal.pbio.1000412) for reporting work involving animal research. Where applicable, authors should refer to any relevant reporting standards documents in this form.

If you have any questions, please consult our Journal Policies and/or contact us: [editorial@elifesciences.org](mailto:editorial@elifesciences.org).

**Sample-size estimation**

- You should state whether an appropriate sample size was computed when the study was being designed
- You should state the statistical method of sample size computation and any required assumptions
- If no explicit power analysis was used, you should describe how you decided what sample (replicate) size (number) to use

Please outline where this information can be found within the submission (e.g., sections or figure legends), or explain why this information doesn’t apply to your submission:

Due to the exploratory nature of the study, no sample-size estimation was done.

**Replicates**

- You should report how often each experiment was performed
- You should include a definition of biological versus technical replication
- The data obtained should be provided and sufficient information should be provided to indicate the number of independent biological and/or technical replicates
- If you encountered any outliers, you should describe how these were handled
- Criteria for exclusion/inclusion of data should be clearly stated
- High-throughput sequence data should be uploaded before submission, with a private link for reviewers provided (these are available from both GEO and ArrayExpress)

Please outline where this information can be found within the submission (e.g., sections or figure legends), or explain why this information doesn’t apply to your submission:

All experiments were performed once.

For sorting of peptide-specific CFSElow CD4 T cells, two technical replicates were included and only those TCR sequences that occurred in both replicates were retained for further analysis.

No data were excluded from the analysis.

**Statistical reporting**

- Statistical analysis methods should be described and justified
- Raw data should be presented in figures whenever informative to do so (typically when N per group is less than 10)
- For each experiment, you should identify the statistical tests used, exact values of N, definitions of center, methods of multiple test correction, and dispersion and precision measures (e.g., mean, median, SD, SEM, confidence intervals; and, for the major substantive results, a measure of effect size (e.g., Pearson's r, Cohen's d)
- Report exact p-values wherever possible alongside the summary statistics and 95% confidence intervals. These should be reported for all key questions and not only when the p-value is less than 0.05.

Please outline where this information can be found within the submission (e.g., sections or figure legends), or explain why this information doesn’t apply to your submission:

1. The Wilcoxon rank sum test and the Wilcoxon signed rank test were used to compare two or more groups, with unpaired and paired analysis as necessary. Bonferroni correction was applied when multiple comparisons were made.

2. The nonparametric Spearman's rank-order correlation was used to test for correlation.

3. The two-sided Fisher’s exact test was used to evaluate the significance of relationship between early/late-converters and CMV, EBV or HSV seropositivity.

Information related to statistical reporting are available in “**Statistics and data visualization**” section in “**Methods**”.

(For large datasets, or papers with a very large number of statistical tests, you may upload a single table file with tests, Ns, etc., with reference to sections in the manuscript.)

**Group allocation**

- Indicate how samples were allocated into experimental groups (in the case of clinical studies, please specify allocation to treatment method); if randomization was used, please also state if restricted randomization was applied
- Indicate if masking was used during group allocation, data collection and/or data analysis

Please outline where this information can be found within the submission (e.g., sections or figure legends), or explain why this information doesn’t apply to your submission:

No group allocation was necessary as all vaccinees were recruited as healthy adults. Blood samples were obtained from a total of 34 healthy individuals that were recruited to the study after it was confirmed that they had no history of a natural HBV infection or prior HBV vaccination (confirmed via both vaccination history and a negative anti-HBs antibody assay)

Samples were randomized for ex vivo CD4 T cell assay between the three groups in the cohort (early, late and non-converters) to minimize batch effects. No blinding protocol was applied. All samples were processed by the same operators.

**Additional data files (“source data”)**

- We encourage you to upload relevant additional data files, such as numerical data that are represented as a graph in a figure, or as a summary table
- Where provided, these should be in the most useful format, and they can be uploaded as “Source data” files linked to a main figure or table
- Include model definition files including the full list of parameters used
- Include code used for data analysis (e.g., R, MatLab)
- Avoid stating that data files are “available upon request”

Please indicate the figures or tables for which source data files have been provided:

The sequencing data that support the findings of this study have been deposited on Zenodo (<https://doi.org/10.5281/zenodo.3989144>). Flow Cytometry Standard (FCS) data files with associated FlowJo workspaces are deposited at flowrepository.org under the following experiment names: epitope mapping: <https://flowrepository.org/id/FR-FCM-Z2TN>; *in vitro* T cell expansion: <https://flowrepository.org/id/FR-FCM-Z2TM>; *ex vivo* CD4 T cell assay: <https://flowrepository.org/id/FR-FCM-Z2TL>

This experiment has been locked for reviewers' access and may be accessed via the following URL:

1. epitope mapping: <https://flowrepository.org/id/FR-FCM-Z2TN> (exclusive access via <https://flowrepository.org/id/RvFr0MDnAlu9yTfdyqxlRL7V9FmgKpD9xSXUCW0Z2YvEtFTDLSFQTVODXXbU5JGz>)

*2. in vitro* T cell expansion: <https://flowrepository.org/id/FR-FCM-Z2TM> (exclusive access via <https://flowrepository.org/id/RvFrcQqCLNR1MoMV5yxgQPDNbHym3EkErfpsa2yz1YAg2k21MblF602n1zegm7It>)

*3. ex vivo* CD4 T cell assay: <https://flowrepository.org/id/FR-FCM-Z2TL> (exclusive access via https://flowrepository.org/id/RvFrzdulKzJvxVHNxCOyXpFGkNUpCW4ahfY6VlXXoKlJg0hwT8YZ5e7OM7Jgp9ru).

Code used for data analysis is available on github <https://github.com/pmeysman/HepBTCR>
